# Rehabilitation Program Using Sensory Integration Therapy for Dropped Head Syndrome: A Case Report

**DOI:** 10.7759/cureus.74929

**Published:** 2024-12-01

**Authors:** Maremi Mizuno, Akio Sakamoto, Ryuichi Nishimura

**Affiliations:** 1 Rehabilitation, Kyoto Karasuma Hospital, Kyoto, JPN; 2 Orthopaedic Surgery, Kyoto University, Kyoto, JPN; 3 Orthopaedic Surgery, Kyoto Karasuma Hospital, Kyoto, JPN

**Keywords:** cervical spine, dropped head syndrome, kyphotic deformity, rehabilitation, sensory integration

## Abstract

Dropped head syndrome (DHS) is characterized by a correctable cervical kyphotic deformity due to weakened cervical paraspinal muscles. Currently, there is no established treatment for the condition. Sensory integration pertains to the processing, integrating, and organizing of sensory information from both the body and the environment. Sensory integration therapy can be incorporated into a rehabilitation program for individuals displaying deficits in the sensory integration process and/or balance. The rehabilitation program had a sensory integration component, including exposure to sensory stimulation for the feet and eyes, and a physical component, involving cervical extensor muscle strengthening exercises incorporating the cervical paraspinal muscle exercises. A 68-year-old man was referred to our institution with DHS. His condition was prominent during standing and walking. A 12-week rehabilitation program was devised, which required four weeks of hospitalization, an outpatient period, and hospitalization for rehabilitation. The rehabilitation program, which included both sensory integration and muscle strengthening, was undertaken by a prior patient with idiopathic DHS. This program attenuated muscle weakness of the cervical extensor muscles, such that the patient was able to look at the ceiling in a sitting posture. In this case of DHS, the rehabilitation program was useful and suggested DHS can be a disorder of deficits in sensory integration and improves when these are addressed.

## Introduction

Dropped head syndrome (DHS), also referred to as chin-on-chest deformity, presents as a cervical kyphotic deformity that results from a weakening of the cervical paraspinal muscles [1,2]. Cervical orthosis keeps the cervical spine in an extended position and improves problems associated with neck discomfort, pain, eating, and social interaction. DHS can develop for a variety of reasons, including Parkinson’s disease and myasthenia gravis, in addition to idiopathic DHS [3]. According to previous studies, these primary diseases are categorized as neurological, neuromuscular, muscular, or other causes [4]. Orthoses and rehabilitation are first-line conservative therapies for DHS, as they address the discomfort and limitations to movement [5,6].

The benefits of a rehabilitation program for DHS have been reported. The aim of this program, known as SHAiR (Short and Intensive Rehabilitation), was to improve the function of the cervical extensors and flexors, as well as restore overall spinal alignment [7]. Improvement in the ability to maintain a horizontal gaze and a reduction in cervical pain occurred rapidly for all five patients in the SHAiR group, which contrasted with the absence of any improvement among the patients in the control group. Rehabilitation for DHS was considered effective, not only in terms of localized exercises, such as those training cervical extensor muscle function, but also for exercises aimed at improving thoracolumbar posture and strengthening the psoas muscles [7].

The SHAiR program aims to “re-establish intentional muscle contractions targeting the spine,” while sensory integration therapy seeks to induce reflexive and responsive actions in the spine. Sensory integration refers to the processing, integration, and organization of sensory information from the body and the environment [8]. Both an excess and deficiency of sensory information, as well as the inability to interpret muscle strength, extent, and/or direction of action, can result in various disorders. Treatment using the sensory integration method involves providing a variety of strong stimuli and working on retraining the body's sensory interpretation of the environment. Exercises that stimulate the deep sensory system and a sense of balance can be used to treat imbalances in sensory integration [8]. Idiopathic scoliosis is more likely to be caused by muscle weakness in the paravertebral and spinal muscles than by sensory issues. However, long-term sensory and balance problems could reduce motor function, potentially leading to atrophy [8,9].

The sensory integration program is a rehabilitation program developed to improve idiopathic DHS. This report presents a case in which the sensory integration program was evaluated.

## Case presentation

**Figure 1:**
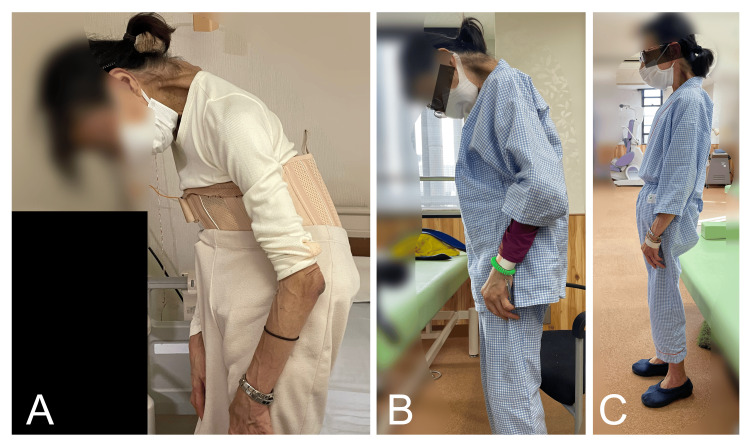
A 68-year-old female patient with DHS The patient’s posture before physical therapy (A), one week after the start of physical therapy (B), and four months after the start of physical therapy at the end of here entire rehabilitation program (C). DHS, dropped head syndrome

**Figure 2:**
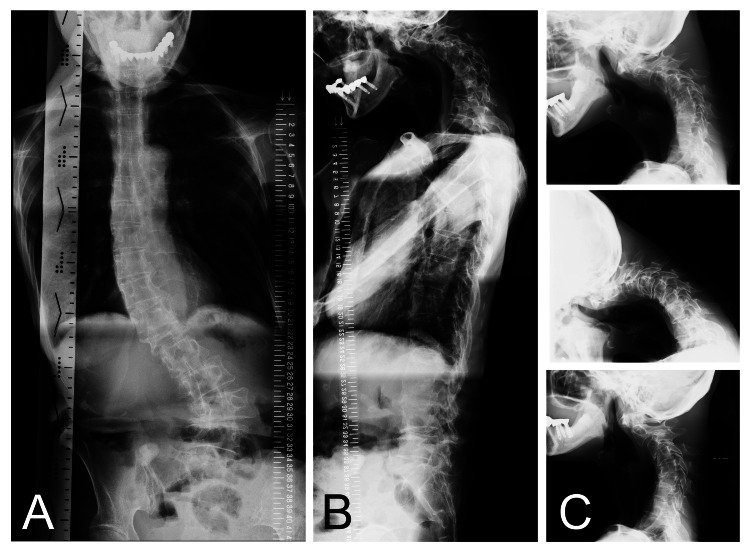
Plain radiographs of the spine of the same patient The view of the entire spine shows scoliosis in the antero-posterior view (A) and kyphosis in the cervical spine in the lateral view (B). The neutral position (C-top), flexion (C-middle), and extension (C-bottom) of the cervical spine are shown.

A 68-year-old female patient was referred to our institute with DHS and difficulty walking. The patient’s height was 150 cm, her weight was 32.1 kg, and her body mass index (BMI) was 14.3 kg/m^2^ (i.e., underweight category). When aged 50 years, the patient was diagnosed with mild depression and has been on medication since then, with her symptoms remaining stable. There were no other specific underlying diseases. Parkinson's disease and myasthenia gravis were ruled out. She had noticed neck flexion approximately nine years ago (when 59 years old) and the symptoms had gradually worsened. Difficulty walking occurred because of an inability to maintain a level gaze 10 months before her rehabilitation (when 67 years old). The symptoms worsened significantly and impacted her daily life three months before rehabilitation commenced. At the initial assessment, she was unable to raise her head and gaze horizontally without assistance and her chin remained in contact with her chest wall. Thus, the patient needed assistance because she could not look straight ahead when walking (Figure 1). Pain in her neck and lower back increased with walking and when standing to cook. The patient’s head drop caused eating difficulties because she could not see what food was on the table in front of her or scoop up food using a spoon. She also had to resort to using a straw to drink. On initial physical examination, her dropped head was easily lifted by others, suggesting that joint contracture of the cervical spine had not yet occurred. The patient’s neck extension muscle strength, measured using manual muscle testing, was grade 2, which was insufficient for lifting her head unaided against gravity. Besides her neck extensors, her other muscles were normal (grade 5) or almost normal (grade 4). The plain radiographs of the patient’s cervical spine revealed kyphosis. The curvature of the cervical spine was determined by the angle formed by lines drawn from the C2 and C7 vertebral endplates on a lateral view. This angle was -74 degrees in the neutral position, -90 degrees in flexion, and -60 degrees in extension. A normal range in the neutral position is between 20 and 40 degrees. Scoliosis was noted in the lumbar spine and dextrodeformity was evident at the first lumbar vertebra (L1). There was no reported history of injury and no associated pain due to the L1 deformity (Figure 2).

**Figure 3:**
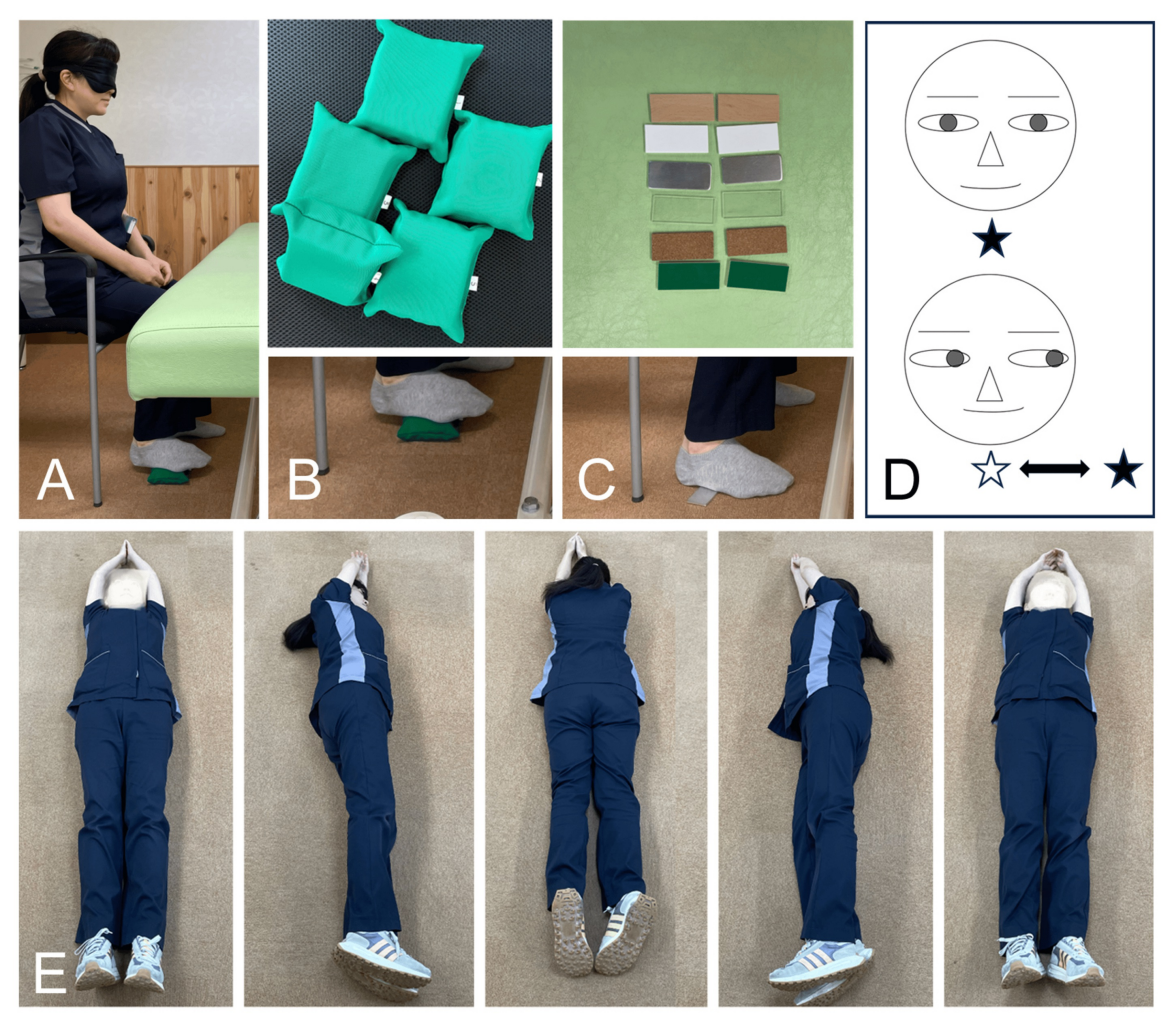
Illustrations of the sensory input procedure Sensory stimulation on the sole (A-C). Discrimination training with sponges of different hardness (B-top) and plates of different materials (C-top) are shown. Eye movements to the target are shown (D). Demonstration of the rolling exercise (E).

**Figure 4:**
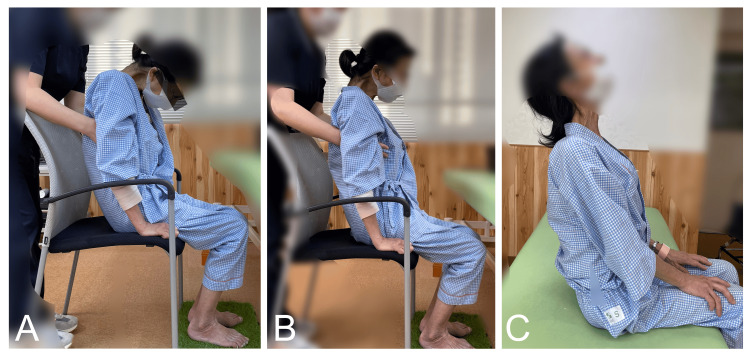
The same patient with DHS Neck extension was unable to be performed at the start of physical therapy (A). A neck extension exercise was performed with assistance; the weight of the upper limbs was supported by a physical therapist during the rehabilitation (B). Cervical spine extension was performed independently by the patient within four months after the start of physical therapy (C). DHS, dropped head syndrome

A detailed summary of the rehabilitation schedule is shown in Table 1. The sensory input procedures are illustrated in Figure 3. In the rehabilitation program, during the first four weeks of hospitalization, rehabilitation therapy was performed once daily for 40 minutes on weekdays. The goal of the exercises for the first four weeks was to improve physical strength and achieve slight voluntary neck movement. This rehabilitation consisted of neck exercises while the patient's upper extremities were supported by a physical therapist. In addition, the patient was instructed to relax her shoulder girdle and thorax muscles. Sensory input was delivered to stimulate the soles of the feet and eye movement [10,11]. In the current case, after the first four-week period, muscle contraction was confirmed. The goal of the exercises in the second four-week period was to lift the head independently and extend the holding time. During that period, rehabilitation therapy was conducted once weekly for 40 minutes in an outpatient setting. The goal of the exercises during the third or last four-week period was to stabilize movement. At that time, the patient gained enough strength to be able to lift her own head without assistance (Figure 1). During the final four weeks of rehabilitation, the patient was re-hospitalized and again received rehabilitation therapy once daily for 40 minutes. Log rolling exercises were performed three times with three rotations on each side. During the third week of the last four-week period of rehabilitation, the patient was able to look at the ceiling while seated (Figure 4). At the end of the rehabilitation program, her neck muscle strength during neck extension went from grade 2 to grade 3. The patient was able to support her neck in both standing and seated positions and keep her head elevated without assistance and maintain an upward gaze toward the ceiling while seated for about five minutes.

**Table 1:** Rehabilitation schedule

Objective	Introduction	Progression	Stabilization
Period	Weeks 1-4	Weeks 5-8	Weeks 9-12
Exercise goal	Improve physical strength, move neck slightly on own	Lift head independently, extend holding time	Stabilize movement
Setting	Inpatient	Outpatient	Inpatient
Frequency	Once daily on weekdays	Once weekly	Once daily on weekdays
Duration	40 minutes	40 minutes	40 minutes
Details	Neck exercises (with upper limb support), relaxation of shoulder girdle and thoracic muscles, sensory stimulation to the soles of feet and eye movement	Same as left	Turning movement (three rotations on one side, performed three times), practice lifting head, and looking at ceiling (in seated position)
The current case	Confirmation for muscle contraction	Patient can lift head independently	Ability to look at ceiling while seated, can eat independently

Three months after the rehabilitation program ended, it became difficult for the patient to hold her head up while walking, but she was still able to lift her head independently and maintained this function during her daily life. Even one year after completing rehabilitation, she was able to lift her head, look up at the ceiling, and hold this position for three minutes. She was also able to eat independently without difficulties, such as spilling food, during meals. She had been attempting to retain her neck muscle strength, although no specific medical rehabilitation was provided since the initial one. She was satisfied with her improvement thus far and declined to participate in additional rehabilitation efforts.

## Discussion

The concept of sensory integration is based on the idea of increasing synaptic responses. This means that when one neuron fires repeatedly to provide input, or when another neuron fires in response to additional inputs from other neurons, the synapse that facilitated this activity is strengthened. The eyes, cutaneous pressure receptors, proprioceptors in muscles, tendons, and joints, as well as the inner ear all contribute to sensory receptivity for posture and movement [12]. When rehabilitating motor skills, repetition and stimuli arising from or in relation to activity manually delivered by a physical therapist's hand can provide the stimuli necessary for the formation of cellular glomeruli. Therapy associated with sensory integration aims to process both endogenous and exogenous stimulation in a coordinated manner. Sensory integration therapy is widely used for children with developmental disorders and has been increasingly used for healthy adults in recent years [13].

For activities of daily living such as walking and maintaining posture, various movements are executed by gathering information from the external environment through visual, auditory, vestibular, and proprioceptive senses. Approaches targeting visual input, vestibular senses, and proprioceptive senses are prominent in proposed sensory integration therapy [14]. Within the sensory system, integration is primarily coordinated by the vestibular organ, the deep sensation system, the visual system, and the superficial sensing system. Spinal disorders can arise due to deficits in sensory integration. As the deficits in sensory integration and balance increase, dynamic and static balance dysfunction adversely affects muscle tone and the occurrence and frequency of a diagnosis of idiopathic scoliosis [8,9].

In the current case of idiopathic DHS, the rehabilitation associated with sensory integration yielded a successful result. The utility of rehabilitation associated with sensory integration in idiopathic scoliosis has been reported [8,9]. The SHAiR program aims to restore voluntary muscle contractions that specifically target spinal stability, whereas sensory integration therapy stimulates each sensory receptor to evoke reflexive and adaptive responses [7]. By combining these distinct approaches, coordinated and timely movement patterns can be effectively stimulated. The sensory integration program incorporated cervical extensor muscle strengthening exercises, including cervical paraspinal muscle exercises. In sensory integration disorders, the goal of treatment is to make sure the body responds to touch normally and to regulate muscle tension. Rehabilitation associated with sensory integration involves helping patients become aware that movement rebuilds sensory integration. Rehabilitation can improve the ability to sense movement and enhance deep sensations, resulting in normal or balanced sensory integration. Eventually, improved sensory integration results in the maintenance of good posture and normal reactions that effectively enhance balance during activities [8,15].

There are several limitations inherent in this case report. Primarily, it is based on one person and there is no control group. A larger sample size and long-term follow-up are necessary. The successful outcome in the current case might be attributed to the patient's specific condition, which featured a severe phase that lasted less than one year. In the current case, the follow-up focused on the patient’s ability to extend her neck, and plain radiographs were not used for follow-up. Further evaluation of plain radiographs is necessary to demonstrate the impact of the rehabilitation program on the cervical spine. She was satisfied with the results of the rehabilitation even one year after her rehabilitation program ended, and additional rehabilitation was declined. However, implementing a short-term program annually could be beneficial for sustaining improved neck posture over the long term.

## Conclusions

In the present case, the effectiveness of the program suggests that DHS may be linked to deficits in sensory integration. Rehabilitation targeting sensory integration can enhance patients' ability to sense movement, regulate muscle tone, and maintain proper posture, ultimately improving motor control and overall physical function. The sensory integration-based rehabilitation program yielded positive results in the treatment of DHS, particularly by improving muscle strength, body alignment, and balance responses. This case emphasizes the potential value of sensory integration-based rehabilitation for managing DHS.
